# Health care supply in patients with Ehlers-Danlos syndromes and generalized hypermobility spectrum disorder: a German perspective

**DOI:** 10.1186/s13023-025-03937-4

**Published:** 2025-08-16

**Authors:** Jonas Rauterberg, Marie Hock, Nikolaus Kernich, Arim Shukri, Henning Klapproth, Vanessa Löw, Michaela Henning, Iliana Tantcheva-Poór

**Affiliations:** 1 Department of Dermatology and Venereology, Faculty of Medicine and University Hospital, University of Cologne, Kerpener Str. 62, 50937 Cologne, Germany; 2 Department of Orthopedic and Trauma Surgery, Faculty of Medicine, University Hospital of Cologne, University of Cologne, Cologne, Germany; 3 Institute of Health and Economics and Clinical Epidemiology, University of Cologne, Cologne, Germany; 4 Pain Center, Department of Anesthesiology and Intensive Care Medicine, University of Cologne, Cologne, Germany; 5 Department of Psychosomatics and Psychotherapy, Faculty of Medicine and University Hospital, University of Cologne, Cologne, Germany

**Keywords:** Hereditary connective skin disorders, Genodermatoses, Ehlers-danlos syndromes, Hypermobility spectrum disorder, Generalized hypermobility, Health care supply, Diagnostic odyssey, Rare diseases, Burden of disease

## Abstract

**Background:**

Diagnosing Ehlers-Danlos syndromes (EDS) and EDS-related “hypermobility spectrum disorder” (HSD) is challenging and cutaneous manifestations often serve as indicators of these rare connective tissue disorders. Only limited data exist on the healthcare of EDS/HSD in Germany as specialized services have been missing and a national register is not available.

**Objectives:**

In 2020, a dermatologic-orthopedic EDS outpatient service was initiated at the University Hospital of Cologne, Germany. The objectives of the present survey were to examine the “medical journey” and the disease burden of our patients.

**Methods:**

A pseudonymized paper survey was sent to all adults who were diagnosed with hypermobile EDS (hEDS), classical EDS (cEDS), classical-like EDS (clEDS) or generalized HSD at the EDS Cologne service from December 2021 until May 2023.

**Results:**

Of the 99 participants, 80 were diagnosed with hEDS/HSD, 16 with cEDS and 3 with clEDS. The mean time to diagnosis was 22.0 years (14.5 years for cEDS/clEDS vs. 23.0 years for hEDS/HSD). 24.2% of the participants had a recognized degree of disability of ≥ 50, the average sick leave in the last 3 months was 4.6 days for cEDS/clEDS and 21.3 days for hEDS/HSD. 44.9% of the hEDS/HSD patients reported on having at least four comorbidities compared to 21.1% in the cEDS/clEDS group (*p* = 0.023). At least 15 medical specialties were involved in the diagnostics and treatment of participants with multiple therapeutic modalities.

**Conclusions:**

This is the first study providing an insight on the healthcare supply of EDS/HSD patients in Germany. Participants in our survey had a much longer “diagnostic journey” than the one in previous studies and suffered from high morbidity despite high healthcare utilization. Our results point to the urgent necessity of a better coordinated, multidisciplinary care for patients with these complex genodermatoses including innovative political structures, further research and international networking.

**Supplementary Information:**

The online version contains supplementary material available at 10.1186/s13023-025-03937-4.

## Background

The Ehlers-Danlos syndromes (EDS) represent a group of 13 rare connective tissue disorders clinically characterized by skin hyperextensibility, joint hypermobility and generalized tissue fragility [1]. They are caused by variants in 20 currently known genes involved in the synthesis and processing of the fibrillar collagens I, III and V as well as in the synthesis of other proteins of the extracellular matrix such as tenascin-X [2]. For the most common hypermobile EDS (hEDS), a molecular testing is not available yet.

Traditionally, EDS fall into the field of dermatology having been independently described by the dermatologists Chernogubov (1891), Ehlers (1901) and Danlos (1908) [3, 4]. According to the 2017 International Ehlers-Danlos classification, skin signs serve as important major and minor criteria for all types and, when available, increase the chances of a positive molecular testing [5]. For example, skin hyperextensibility, velvety skin texture and easy bruising belong to the diagnostic criteria for classical and classical-like EDS (cEDS, clEDS); atrophic scarring is present in patients with cEDS, but not in clEDS [2]. Five cutaneous symptoms contribute to the diagnosis of hEDS which currently relies only on a set of clinical criteria including symptomatic generalized hypermobility, a systemic score of 12 items, positive family history and the exclusion of other connective tissue disorders [1, 6, 7]. The term “hypermobility spectrum disorder” (HSD) was introduced in 2017 and refers to patients suffering from symptomatic hypermobility, who do not meet the hEDS criteria and in whom other connective tissue disorders have been excluded [6, 7]. Due to overlapping phenotypes and probably the same pathogenesis, hEDS and HSD are increasingly considered one group of disorders [8–11].

Until recently, a centralized Ehlers-Danlos register and specialized centres have been missing in Germany. In 2020, we established at the University Hospital of Cologne a weekly dermatologic-orthopedic service for EDS patients. To better understand the needs of our patients, we conducted an anonymous survey including all adults with symptomatic generalized hypermobility who were diagnosed with hEDS, cEDS, clEDS or HSD. The objectives of our study were (1) to examine the diagnostic journey and healthcare supply of the participants; (2) to assess their burden of disease and (3) to study respondents’ mental comorbidities in correlation to pain. Here, we present our results regarding the first two objectives.

## Materials and methods

### Study design

This cross-sectional epidemiological study was conducted as a single-centre study between December 2021 and May 2023, as part of the EDS Cologne outpatient service. A pseudonymized paper survey was sent to all adults who consented and were diagnosed with hEDS, cEDS, clEDS or generalized HSD. All patients presented with generalized hypermobility as defined according to the 2017 hEDS criteria, i.e. having a Beighton score of ≥ 5 for pubertal men and women up to the age of 50 and ≥ 4 for women over 50 years old [1]. All patients showed musculoskeletal complaints in the context of a so-called “symptomatic hypermobility” due to joint instability [2, 12]. Exclusion criteria included minors, an unexplained connective tissue disease or EDS not listed above, inadequate understanding of the German language and/or a cognitive impairment. As hEDS and HSD are increasingly perceived as one entity, hEDS and HSD were pooled in similarity to recent studies for analysis [8–11]. Due to the relatively small number of patients with cEDS and clEDS, these patients were assessed as so-called “monogenetic EDS”.

The study was approved by the Ethics Committee of the University Hospital of Cologne, Germany, in accordance with the principles of the Declaration of Helsinki (registered as DRKS00024554).

### Instruments

Demographic and clinical data were collected using a self-developed questionnaire that captured socio-demographic characteristics (age, sex, education, employment status), diagnostic journey (first symptoms and age of onset, age at diagnosis, alternative diagnoses prior to EDS), current burden of disease (including recognized level of disability, days on sick leave in the last three months, comorbidities) and health care utilization (specialists involved, surgical interventions, physiotherapy, psychotherapy, pain medication and medical rehabilitation). The questionnaire included dichotomous, multiple-choice and open-ended items. Participants could list up to five symptoms and four previous diagnoses as free-text responses. Pharmacological treatments were also reported in free-text and categorized by substance group and usage pattern (“daily” vs. “as needed”). Satisfaction with treatment modalities was assessed using a 5-point Likert scale. Further details regarding the questionnaire are provided in Additional file 1.

Participants were also provided with instruments to assess pain (*Deutscher Schmerzfragebogen* [DSF]), mental comorbidity (*Patient Health Questionnaire*– German version [PHQD]), sleep quality (*Pittsburgh Sleep Quality Index* [PSQI]), and fatigue (*European Organisation for Research and Treatment of Cancer Quality of Life Questionnaire*– Fatigue Module [EORTC QLQFA12]). The analysis of these instruments is discussed elsewhere.

### Statistical analysis

A detailed overview on the sample size calculation is given in the Additional file 1. Descriptive statistics for all socio-demographic and clinical variables were analyzed for the entire sample and for two subgroups monogenetic (cEDS/clEDS vs. hEDS/HSD). Categorical variables are reported as absolute numbers and percentages, while continuous variables are presented as medians with interquartile ranges (IQRs) and additionally described by means with standard errors (SE). Total prevalences were calculated with 95% confidence intervals (CIs) using the Clopper-Pearson method for categorical variables. To account for multiple testing, group differences between monogenetic EDS and hEDS/HSD were assessed only for selected categorical variables (symptoms of disease manifestations, other diagnoses prior to EDS/HSD, number of patients with comorbidities) using Fisher’s exact test, and for selected continuous variables (age at first manifestation, age at EDS/HSD diagnosis, average time to diagnosis) using the Mann–Whitney U test. No imputation was performed for missing data. Data were analyzed using IBM^®^ SPSS^®^ Statistics for Mac OS, version 28.0.1.1(14) (IBM Corp: Armonk, New York). For all statistical tests, a *p* < 0.05 was considered statistically significant.

## Results

### Demographic information

Of the 132 adults who fulfilled the inclusion criteria and consented to the survey, there were 99 respondents whose data were further analyzed (total response rate 75.0%). The demographics are presented in Table 1. Most of the participants were diagnosed with hEDS/HSD (80.8%); 16 patients (16.2%) were diagnosed with cEDS and three with clEDS.

**Table 1:** Demographic characteristics of monogenetic EDS and hEDS/HSD patients

Characteristics	Total	Monogenetic	hEDS/HSD
	*n* (%)	*n* (%)	*n* (%)
**EDS diagnosis**	99 (100)		
**Monogenetic**	19 (19.2)		
Classical EDS	16 (16.2)		
Classical-like EDS	3 (3.0)		
**hEDS/HSD**	80 (80.8)		
hEDS	27 (27.3)		
HSD	53 (53.5)		
**Age**			
Years, mean (SD)	35.9 (12.9)	36.4 (16.3)	35.8 (12.1)
**Sex**			
Female	87 (87.9)	16 (84.2)	71 (88.8)
Male	10 (10.1)	3 (15.8)	7 (8.8)
Diverse	2 (2.0)	-	2 (2.5)
**Education**			
Lower secondary education	23 (23.2)	6 (31.6)	17 (21.3)
Upper secondary education/ General university entrance qualification	47 (47.5)	9 (47.4)	38 (47.5)
Bachelor or equivalent	29 (29.3)	4 (21.1)	25 (31.3)
**Employment**			
Employed	65 (65.7)	15 (78.9)	50 (62.5)
Non-employed	9 (9.1)	2 (10.5)	7 (8.8)
Sick leave	9 (9.1)	-	9 (11.3)
Disability pension	16 (16.2)	2 (10.5)	14 (17.5)

Note. Monogenetic, includes: classical EDS and classical-like EDS; hEDS, hypermobile Ehlers-Danlos syndrome;

HSD, hypermobility spectrum disorder; Employed, includes: full/part time employment; Non-employed, includes: unemployed, housewife, retired

Of the 99 participants, 67 individuals (67.7%) were diagnosed with EDS/ HSD at our service. The remaining 32 participants (32.3%) had received their diagnosis at other services prior to their presentation at our centre. In all externally diagnosed cases, we verified the diagnosis of EDS/ HSD based on review of the existing medical documentation and clinical reassessment, ensuring consistency with the current diagnostic criteria for hEDS, cEDS, clEDS, and HSD. In the monogenetic group, the diagnosis was molecularly confirmed in 13 out of the 16 cEDS patients and in all three patients with clEDS.

### Diagnostic journey

The diagnostic journey of our patients is presented in Table 2. The median age at disease presentation was 7.0 years for the entire cohort, with an earlier onset observed in the monogenetic subgroup (5.0 years for cEDS/clEDS vs. 10.0 years for hEDS/HSD, *p* = 0.023). The patients were diagnosed with EDS/HSD at a median age of 33.0 (IQR 22.0) years, with a median of 20.5 (IQR 25.5) years for cEDS/clEDS and 34.0 (IQR 19.0) years for hEDS/HSD (*p* = 0.001). Almost half of the participants (44.9%) have received an alternative diagnosis before. Additionally, 63.6% of participants used other sources - such as social media and patient support groups - to confirm their diagnosis and/ or obtain further information.

**Table 2:** The diagnostic journey of monogenetic EDS and hEDS/HSD patients in our cohorts

		Total			Monogenetic		hEDS/HSD		
		*n*	median (IQR)	*p*-value*	*n*	median (IQR)	*n*	median (IQR)	
**Age of manifestation**,** years** (mean 10.30 [SE 0.8])		98	7.0 (10.0)	0.017	19	5.0 (5.0)	79	10.0 (10.0)	
**Age of EDS/ HSD diagnosis**,** years** (mean 32.3 [SE 1.3])		98	33.0 (20.0)	0.001	19	20.5 (25.5)	79	34.0 (19.0)	
**Average time to the EDS/ HSD diagnosis**,** years** (mean 22.9 [SE 1.2])		97	22.0 (18.0)	0.002	18	14.5 (12.75)	79	23.0 (17.0)	
		**n**	**%**	**95% CI**	**n**	**%**	**n**	**%**	
**Symptoms of disease manifestations** ^a^		94	100		19	100	75	100	
Musculoskeletal pain		33	35.1	[25.5, 45.6]	5	26.3	28	37.3	
Joint instability		42	44.7	[34.4, 55.3]	6	31.6	36	48.0	
Cutaneous manifestations		10	10.6	[5.2, 18.7]	7	36.8	3	4.0	
Others		9	9.6	[4.5, 17.4]	1	5.3	8	10.7	
**Other diagnoses prior to EDS/ HSD**		98	100		19	100	79	100	
Patients who received other diagnoses		44	44.9	[34.8, 55.3]	4	21.1	40	50.6	
Most common other diagnoses									
Orthopedic disorders		22	50.0	[34.6, 65.4]	2	50.0	20	50.0	
Somatization disorders		11	25.0	[13.2, 40.3]	-		11	27.5	
Fibromyalgia		7	15.9	[6.6, 30.1]	-		7	17.5	
Collagenosis		4	9.1	[2.5, 21.7]	2	50.0	2	5.0	
**The provisional diagnosis of EDS/ HSD was made by** ^b^:		99	100		19	100	80	100	
The attending physician		66	66.7	[56.5, 75.8]	13	68.4	53	66.3	
Orthopedist		20	30.3	[19.6, 42.9]	2	14.3	18	34.6	
Human geneticist		12	18.2	[9.8, 29.6]	3	21.4	9	17.3	
General practitioner		10	15.2	[7.5, 26.1]	1	7.1	9	17.3	
Rheumatologist		6	9.1	[3.4, 18.7]	2	14.3	4	7.7	
Dermatologist		5	7.6	[2.5, 16.8]	3	21.4	2	3.8	
Internist		3	4.5	[0.9, 12.7]	-		3	5.8	
Pediatrician		3	4.5	[0.9, 12.7]	3	21.4	-		
Centre for rare diseases		3	4.5	[0.9, 12.5]	-		3	5.8	
Neurologist		2	3.0	[0.4, 10.5]	-		2	3.8	
Surgeon		2	3.0	[0.4, 10.5]	-		2	3.8	
**Other sources used to confirm the EDS/ HSD Diagnosis**		99	100		19	100	80	100	
Patients who used other sources to confirm the diagnosis		63	63.6	[53.4, 73.1]	6	31.6	57	71.3	
Internet ^b^		50	50.5	[40.3, 60.7]	5	26.3	45	56.3	
Patient support group ^b^		34	34.3	[25.1, 44.6]	4	21.1	30	37.5	
Others ^b^		22	22.2	[14.5, 31.7]	2	10.5	20	25.0	

Note. hEDS, hypermobile Ehlers-Danlos syndrome; HSD, hypermobility spectrum disorder; CI, confidence interval; IQR, interquartile range; SE, standard error

^a^ Symptoms of disease manifestations, recall of key symptoms as free text, which was coded. Numbers do not sum to the total because of missing data;

Joint instability, includes: joint hypermobility, subluxations / dislocations; Cutaneous manifestations, include: skin vulnerability, prolonged wound healing, easy bruising, skin hyperextensibility, Others symptoms, include: hallux valgus, hips dysplasia, prolaps, arthrosis, dental problems, blue sclera; ^b^ Multiple references may occur; totals do not add up to 100%. Other sources, includes family and relatives. *p-values calculated based on the Mann-Whitney U test for group comparisons (differences in medians) between monogenetic and hEDS/HSD diagnoses

### Burden of disease and comorbidities

Almost half of the respondents had a recognized degree of disability, with 24.2% of them having a disability of 50 or greater. Applications for severe disability or upgrading were submitted by 22 participants, with no significant differences between the subgroups. Additionally, about one-third of participants reported that their daily functioning was impaired by pain over the last three months (Table 3). For both subgroups, pain (60.2%) and orthopedic problems (joint instability 38.8%, other orthopedic disorders 36.7%) were reported as “severely affecting daily activities”, followed by gastrointestinal complaints (35.7%) and fatigue (32.7%) (Additional file 2).

**Table 3:** Patient-reported health and social care utilization; burden of disease

	Total		Monogenetic	hEDS/ HSD
Parameter	*n* (%)	95% CI**	*n* (%)	*n* (%)
	99 (100)		19 (100)	80 (100)
**Health-care providers involved in the diagnostic process or management process** ^*^				
Patients seen by				
Orthopedist	92 (92.9)	[86.0, 97.1]	17 (89.5)	75 (93.8)
General practitioner	91 (91.9)	[84.7, 96.4]	17 (89.5)	74 (92.5)
Physiotherapist	63 (63.6)	[53.4, 73.1]	15 (78.9)	48 (60.0)
Internist	62 (62.6)	[52.3, 72.1]	10 (52.6)	52 (65.0)
Neurologist	55 (55.6)	[45.2, 65.5]	6 (31.6)	49 (61.3)
Dermatologist	53 (53.5)	[43.2, 63.6]	9 (47.4)	44 (55.0)
Human geneticist	46 (46.5)	[36.4, 56.8]	12 (63.2)	34 (42.5)
Dentist	41 (41.4)	[31.6, 51.8]	4 (21.1)	37 (46.3)
Ophthalmologist	38 (38.4)	[28.8, 48.7]	8 (42.1)	30 (37.5)
Psychotherapist	37 (37.4)	[27.9, 47.7]	7 (36.8)	30 (37.5)
Surgeon	33 (33.3)	[24.2, 43.5]	7 (36.8)	26 (32.5)
Pain therapist	33 (33.3)	[24.2, 43.5]	5 (26.3)	28 (35.0)
Gynecologist	33 (33.3)	[24.2, 43.5]	3 (15.8)	30 (37.5)
Otorhinolaryngologist	24 (24.2)	[16.2, 33.9]	1 (5.3)	23 (28.8)
Psychiatrist	23 (23.2)	[15.3, 32.8]	2 (10.5)	21 (26.3)
Urologist	21 (21.2)	[13.6, 30.6]	3 (15.8)	18 (22.5)
**Treatment Modalities**				
Physical Therapy	94 (94.9)	[88.6, 98.3]	18 (94.7)	76 (95.0)
Types of physical therapy				
Active & Passive	64 (66.7)	[56.3, 76.0]	9 (50.0)	55 (70.5)
Active ^a^	7 (7.3)	[3.0, 14.4]	2 (11.1)	5 (6.4)
Passive ^b^	20 (20.8)	[13.2, 30.3]	6 (33.3)	14 (17.9)
Long-term therapy	60 (67.4)	[56.7, 77.0]	11 (73.3)	49 (66.2)
Instructions for home exercises	84 (84.8)	[76.2, 91.3]	16 (84.2)	68 (85.0)
Use of orthoses/ splints	66 (66.7)	[56.5, 75.8]	12 (63.2)	54 (67.5)
Psychotherapy	68 (68.7)	[58.6, 77.6]	12 (63.2)	56 (70.0)
Rehabilitation Programms	47 (47.5)	[37.3, 57.8]	8 (42.1)	39 (48.8)
Surgical Interventions ^c^, median (IQR)	5.0 (6.5)		3.0 (6.0)	5.0 (7.0)
Multimodal Pain Therapy	44 (44.4)	[34.5, 54.8]	8 (42.1)	36 (45.0)
**Pain Pharmacotherapy**				
Non-opioid	81 (83.5)	[74.6, 90.3]	14 (73.7)	67 (85.9)
daily	46 (47.4)	[37.2, 57.8]	10 (52.6)	36 (46.2)
on demand	63 (64.9)	[54.6, 74.4]	8 (42.1)	55 (70.5)
Opioids	15 (15.5)	[8.9, 24.2]	1 (5.3)	14 (17.9)
daily	11 (11.3)	[5.8, 19.4]	1 (5.3)	10 (12.8)
on demand	4 (4.1)	[1.1, 10.2]	-	4 (5.1)
**Degree of disability**				
Degree of disability ≥ 20, n (%)	24 (24.2)	[16.2, 33.9]	5 (26.3)	19 (23.8)
Severely disabled patients (Degree of disability ≥ 50), n (%)	24 (24.2)	[16.2, 33.9]	6 (31.6)	18 (22.5)
**Days on Sick Leave (last 3 months)**, median (IQR)	3.0 (19.5)		0.0 (5.0)	5.0 (25.0)
**Impairment due to pain (last 3 months)**^**d**^, median (IQR), days	5.0 (55.0)		10.0 (28.0)	15.0 (54.0)

Note: CI: confidence interval; hEDS, hypermobile Ehlers-Danlos syndrome; HSD, hypermobility spectrum disorder; Long-term therapy: duration of physical therapy over a period > 1year

^a^ Only active therapy; ^b^ Only passive therapy; ^c^ Surgical Interventions Range: 0–90; ^d^ Impairment due to pain in the last 3 months (at work, at school, at home). Surgical Interventions, mean 7.5, SE [1.2], Days on Sick Leave (last 3 months), mean 18.0, [3.2], Impairment due to pain (last 3 months), mean 29.4, [3.3]

**Confidence intervals calculated using the Clopper-Pearson method for categorical variables

Missing data: Physical Therapy Type *n* = 3, Long-term therapy *n* = 10, Pain Pharmacotherapy *n* = 2

Surgical Interventions *n* = 6, Days on Sick Leave in the last 3 months *n* = 3, Impairment due to pain in the last 3 months *n* = 9

Most participants (78.6%) reported having an additional diagnosis alongside EDS, including asthma (21.4%), depression (21.4%), irritable bowel syndrome (14.3%), and postural orthostatic tachycardia syndrome (13.3%) (Additional file 3). The percentage of patients with comorbidities in the hEDS/HSD group was higher than in the monogenetic group (86.1% vs. 47.4%, respectively, *p* < 0.001). Furthermore, half of the hEDS/HSD patients (44.9%) reported having at least four comorbidities, compared to 21.1% in the monogenetic group (*p* = 0.023).

### Health care utilization

Participants reported that at least 15 different medical specialties were involved in the diagnostic process and management of their symptoms (Table 3). The median number of surgical interventions in our cohort was 5.0 (IQR 6.5), ranging from 0 to 90 surgeries. Of the 85 participants who reported a surgical intervention, 52 (57.8%) were due to orthopedic indications. Almost all patients (94.9%) received some physical therapy, and 67.4% reported on undergoing long-term physical therapy (for the purpose of this study, the latter was defined as “physical therapy over a period of more than one year”). In 66.7% of cases, both active and passive physiotherapy interventions were applied. Most patients required pain pharmacotherapy including non-opioids (83.5%) and opioids (15.5%). Half of the respondents received multimodal pain treatment (44.4%) and/or participated in a medical rehabilitation program (47.5%). Moreover, 68.7% received psychotherapy (Table 3).

## Discussion

In our survey, all respondents showed generalized symptomatic hypermobility and were diagnosed with hEDS, cEDS, clEDS or HSD according to the 2017 EDS classification. For further analysis, we divided our cohort into two groups: cEDS/clEDS vs. hEDS/HSD. Over 80% of participants belonged to the hEDS/HSD subgroup and were primarily females, well-educated and on average 36 years old. These demographics are in accordance with previous studies showing that hEDS constitutes 80 to 90% of all EDS and that up to 80% of the hEDS/HSD patients are females [6, 13–16]. This gender bias has been attributed to hormonal changes causing more severe symptoms in women starting puberty [2, 16, 17] and points to a polygenic pathogenesis of hEDS/HSD [2].

### Diagnostic journey and disease manifestation

In our cohort, the median time to a correct diagnosis was 22.0 years (IQR 18.0), with a significantly shorter diagnostic delay observed in individuals with monogenic EDS [median 14.5 years (IQR 12.75)] compared to those with hEDS/HSD [median 22.0 years (IQR 18.0); *p* = 0.002.] Older studies have demonstrated that EDS patients often undergo a diagnostic “odyssey” with a mean length of 14 years [18]. Our numbers are much higher than in a recent questionnaire through the *International Ehlers-Danlos Society* showing an average time to diagnosis of 10.39 years for hEDS [19]. According to a recent EURODIS survey, the diagnostic journey for people living in Europe with a rare disease lasts 4.7 years [20]. Our results are alarming as diagnostic delays correlate with a higher burden of disease and an increased health care utilization in these patients [21]. Prior to receiving an EDS diagnosis, half of respondents were given at least one alternative diagnosis. Orthopedic disorders (50.0%), somatization disorder (25.0%), fibromyalgia (15.9%), and collagenosis (9.1%) were the most common. This prolonged journey might also explain why 63.6% of respondents reported on having used other sources to confirm their diagnosis, as well as the participants’ high educational status. Previous research shows that patients with rare diseases are often urged to act on their own and stay connected via social media [22]. During the COVID pandemic, support groups such as the *International Ehlers-Danlos Society* have grown exponentially fast [23].

The diagnostic “odyssey” of patients with EDS/HSD is to be explained by the lack of awareness about hypermobility among medical professionals, usually an insidious onset of multisystemic, often non-specific symptoms (e.g. pain and diverse orthopedic complaints) and the lack of a laboratory diagnostic test for hEDS/HSD [17, 19, 24–27]. Females and patients aged 2–18 years at symptom onset were shown to have a higher risk for diagnostic delays regarding rare diseases which also applies to our cohort [28, 29]. The absence of German Ehlers-Danlos centres (at least until recently) and of a centralized register is an important contributing factor having left medical professionals in Germany with limited to no training about EDS and hypermobility.

### Disease manifestation

In our survey, the median age of disease onset was reported as 5.0 (IQR 5.0) years for cEDS/clEDS and 10.0 (IQR 10.0) years for hEDS/HSD. For both groups, the most common initial presentations included musculoskeletal pain and joint instability (e.g. repeated injuries and multiple subluxations/dislocations). This is in line with previous studies suggesting that when symptomatic, joint hypermobility manifests in 75% of cases before the age of 15 [6, 17, 30]. Here, it is important to stress that the 2017 hEDS criteria do not apply for patients who have not reached biological maturity and a “Pediatric Hypermobility Framework” has been recently developed [31].

In our cohort, cutaneous manifestations such as skin vulnerability, prolonged wound healing, easy bruising and hyperelasticity were reported as “presenting symptoms” in 36.8% of cEDS/clEDS and in 4.0% of hEDS/HSD patients (Table 2). Dermatologic symptoms are typically more striking in the monogenetic types, possibly contributing to an earlier diagnosis in the cEDS/clEDS group. Patients with cEDS/clEDS show an extreme skin hyperextensibility, soft “doughy” skin and large ecchymoses that are not present in the hEDS/HSD group. In addition, the cEDS patients complain from a severe skin fragility with papyraceous and hemosiderotic scar formation, molluscoid pseudotumors and subcutaneous spheroids [2, 32]. Thus, generalized hypermobility combined with abnormal skin features should serve as a “red flag” especially for the monogenetic EDS types. Accordingly, a recent survey demonstrated a correlation between generalized hypermobility, poor healing, easy bruising, atrophic scars, skin hyperextensibility and developmental dysplasia of the hip with a positive molecular testing in 72 pediatric patients who were suspected of having a monogenetic EDS [33].

### Burden of disease and comorbidities

In our cohort, 24.2% of patients reported on having a degree of disability of at least 50 corresponding to a “severe disability” in Germany. This percentage is much higher than the one of 9,5% in the total German population [34]. The average sick leave in the last 3 months was 4.6 days for the monogenetic group and 21.3 days for the hypermobile group which is again much higher than the average of 15.0 sick days for the German employees as reported for the whole calendar year in 2022 [35]. These results support previous data showing high morbidity in individuals with EDS measured by a lower health-related quality of life (QOL) than in the general population [36]. Diverse comorbidities (i.e. anxiety, depression, pain, fatigue and sleep disorders) and functional disability have been repeatedly demonstrated as negative predictors of QOL in EDS/HSD patients [36–39]. In our survey, almost 80% of respondents had at least one comorbidity and nearly 45% reported on having four or more comorbidities. In a survey of 505 individuals with hEDS the average number of co-diagnoses was 10.45 [19]. Both hEDS and HSD patients show similar comorbidities including fatigue, chronic pain syndrome, mental disorders (e.g. depression and anxiety), vegetative dysautonomia, gastrointestinal and urogenital dysfunction as well as a mast cell activation syndrome [10, 19, 40–47]. Asthma, depression, irritable bowel syndrome and postural tachycardia syndrome (POTS) were the most common comorbidities in our total cohort with pain, gastrointestinal complaints and fatigue being reported as “severely affecting daily activities” (Additional file 2). There is growing evidence suggesting that patients with the monogenetic EDS suffer from similar comorbidities [2, 32, 48]. Further research is required in this direction. Our results also highlight the importance of having a multidisciplinary team that is trained to manage the various manifestations of EDS/HSD, including hypermobility-related comorbidities.

### Health care utilization

Similar to other rare multisystemic diseases, patients with EDS/HSD require a high health care supply including multiple medical visits with different specialists (Table 3). In the absence of an early appropriate care, chronification of complaints makes therapy more difficult and costly [49]. In our survey, participants reported an average of 7.6 surgical interventions, ranging from 0 to 90 surgeries per individual. These numbers are particularly notable in light of a recent survey showing a 91% complication rate for orthopedic surgeries in EDS patients, including persistent pain, continued instability, and poor healing [50]. Most respondents have obtained a pain pharmacotherapy including both opioids and non-opioids, and almost half of respondents were treated using a multimodal pain approach, which confirms previous data stating high usage of opiod and non-opioid medications among patients with EDS [10, 51, 52]. 94.9% of participants have received some physical therapy and 67.4% have received a “long-term” physical therapy that was defined for the purpose of our study as “physical therapy over a period of more than one year”. 68% of respondents have received a psychotherapy during their lifetime and this rate is higher than the self-reported rate by German adults having been diagnosed with a mental disorder (24.5%) [53].

High rates of disability in our cohort despite the high health care utilization point to the necessity of a coordinated care, also involving the primary care physicians and therapists. Early recognition of “hypermobility” with its various presentations would help in reducing unnecessary interventions, chronification of symptoms and negative medical encounters on behalf of those with EDS/HSD. Based on our experience, we agree with previous surveys emphasizing the necessity of specialized services trained to evaluate - at least initially - the patients’ complaints and needs, which are often multisystemic, non-specific and might be severely disabling [8, 14]. As in other pain-associated chronic disorders, a holistic approach including physical, psycho- and pain therapy is currently recommended; it appears to improve both musculoskeletal and systemic presentations such as fatigue, vegetative dysautonomia and psychological distress [14, 51, 54–57]. Patient education regarding the diagnosis and therapeutic options, including life style modifications, is essential and first steps in developing face-to-face and online programs for patients with EDS/HSD have been taken [58, 59]. However, there is only limited evidence on the clinical outcomes of the different interventions making clinical trials, guidelines and patient registers important tasks for the Ehlers-Danlos centres and communities [30].

### Limitations and advantages

Our cohort is relatively small with only 99 participants, 80% of them having the most common hEDS/HSD. Thus, this survey is not representative for all EDS types. As our findings exclusively rely on the participants’ self-reports, they are subject to the respondents’ perception and recall bias. Data from a national registry or health insurance companies would have been more reliable, but are currently - partly due to the strict data protection guidelines regarding the insurance policy in Germany - not available.

Moreover, this is a single-centre study conducted at a specialised university outpatient clinic that may reduce the external validity and generalisability of our results to the broader German EDS/HSD population. On the other hand, this approach ensures high internal validity through a standardised data collection process, thus increasing the reliability of the results especially for hEDS and HSD, which presently remain clinical diagnoses due to the absence of confirmatory tests. Furthermore, our response rate of 75% introduces the potential for non-response bias. As we collected no systematic data on the non-respondents, we cannot rule out the possibility that the survey participants differ from non-respondents in terms of disease severity, age, or other sociodemographic or clinical characteristics.

In conclusion, this is the first study providing information on the health care supply of individuals living with EDS/HSD in Germany. It is worrisome that these patients often witness a diagnostic delay of several decades to obtain the correct diagnosis making their complaints chronic and more difficult to treat. Despite high utilization of health care, EDS/HSD patients have a high burden of disease reflected by a high percentage of disability and sick leave days in our survey. Our study underscores the necessity of a coordinated care with multidisciplinary teams trained in the management of EDS/HSD and further research aiming at the development of causal and evidence-based therapeutic approaches.

## Supplementary Information

Below is the link to the electronic supplementary material.

Supplementary Material 1

Supplementary Material 2

Supplementary Material 3

## Data Availability

The data that support the findings of this study are available from the corresponding author upon reasonable request.
