# Supplementary material for: Health care supply in patients with Ehlers-Danlos syndromes and generalized hypermobility spectrum disorder: a German perspective
Source: Orphanet J Rare Dis. 2025 Aug 16;20:436. doi: 10.1186/s13023-025-03937-4 (PMC12358066; doi:10.1186/s13023-025-03937-4)
Supplement: Supplementary file 1 — Supplementary Material 1 [file 13023_2025_3937_MOESM1_ESM.docx]

# **Health Care Supply in Patients with Ehlers-Danlos Syndrome and Hypermobility Spectrum Disorder: A German Perspective**

## **Supporting information**

## Among the 99 participants included in the analysis, 67 individuals (67.7 %) were diagnosed directly at the EDS Cologne outpatient clinic. The remaining 32 participants (32.3 %) had received their diagnosis externally prior to their presentation at our center. In all externally diagnosed cases, the original diagnosis was verified at our center through review of existing medical documentation and clinical reassessment, ensuring consistency with current diagnostic standards for hEDS, cEDS, clEDS, and HSD.

## **Survey Distribution and Response Rate**

A total of 132 adult patients diagnosed with hEDS, cEDS, clEDS, or G-HSD were considered eligible for the study between December 2021 and May 2023. During their visit to the EDS Cologne outpatient clinic, these individuals were provided with verbal information regarding the study. Thereafter, each of them was sent a paper-based questionnaire along with a written informed consent form via standard postal mail. Participants were requested to return both the completed questionnaire and the signed consent form.

Of the 132 eligible patients, 99 (75%) returned both documents and were included in the final analysis. Among the remaining 33 patients, 31 did not respond and two withdrew.

Upon receipt, the consent forms were immediately separated from the questionnaires, and all survey data were pseudonymised before being entered into the study database. A reminder letter was dispatched to non-responders after eight weeks; however, no further follow-up was conducted. It is important to note that no records were kept regarding the total number of patients who have been verbally informed about the study and refused to participate.

Figure 1 provides a visual summary of the participant flow from eligibility to analysis.

Figure 1. Participant Flow


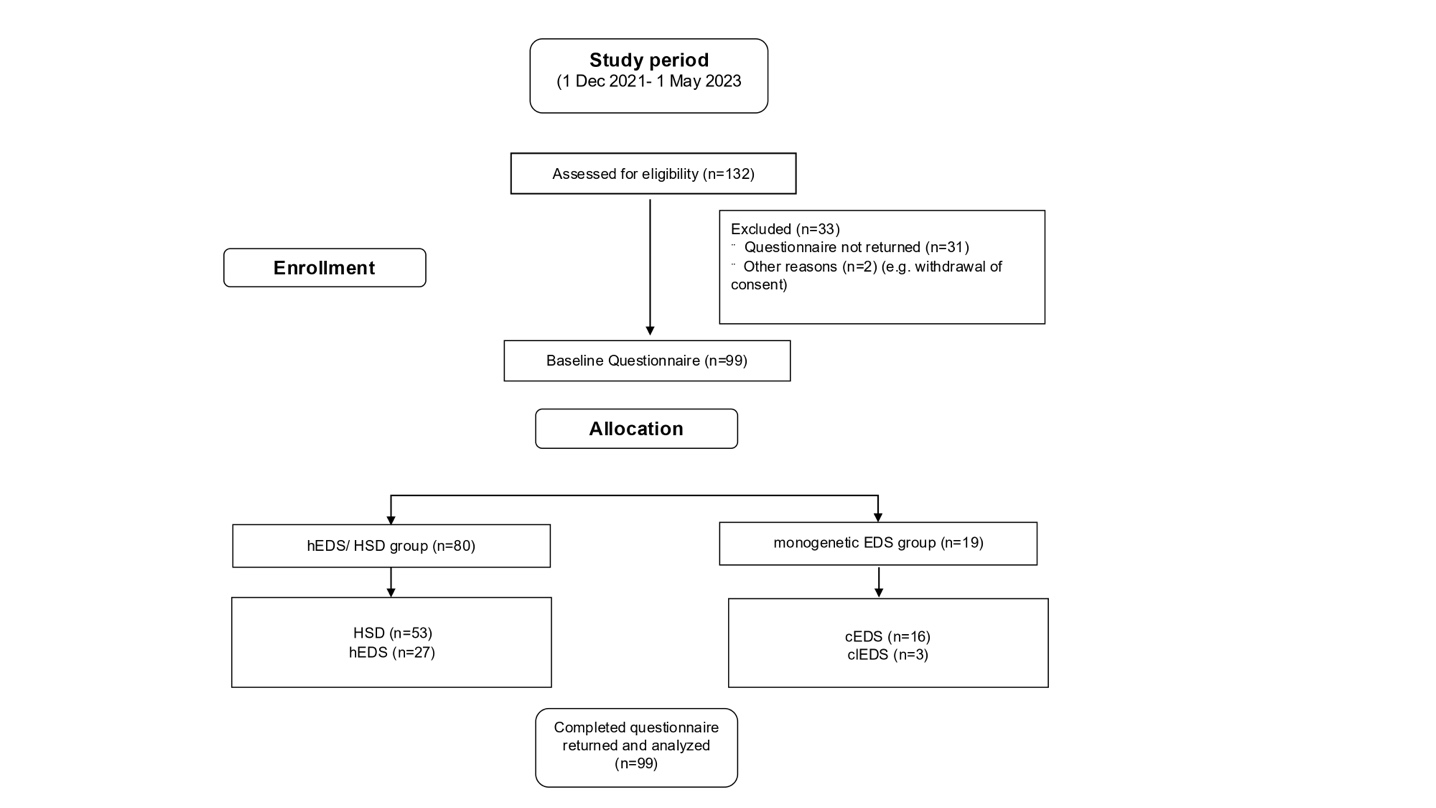


## **The sample size calculation**

The sample size was calculated based on previous studies and their estimation of the proportion of patients with various comorbidities and complaints (such as pain, anxiety, depression, sleep disorders and fatigue). The reported proportions for each comorbidity or complaint range from approximately 10% to approximately 90%. The total number of cases is n=70 to estimate all expected proportions with adequate accuracy.

The following table shows confidence interval limits for case numbers between 20 and 200 and prevalences between 10% and 90%.

| case numbers | | | Prevalence 95%- CI | | |
| --- | --- | --- | --- | --- | --- |
|  | 50% | 60%  40% | 70%  30% | 80%  20% | 90%  10% |
| 20 | 27 – 73 | 36 – 81  19 - 64 | 46 – 88  12 – 54 | 56 – 94  6 – 44 | 68 – 99  1 – 32 |
| 30 | 31 – 69 | 41 – 77  23 – 59 | 51 – 85  15 – 49 | 61 – 92  8 – 39 | 73 – 98  2 – 27 |
| 40 | 34 - 66 | 43 – 75  25 – 57 | 53 – 83  17 – 47 | 64 – 91  9 – 36 | 76 – 97  3 – 24 |
| 50 | 36 - 64 | 45 – 74  26 – 55 | 55 – 82  18 – 45 | 66 – 90  10 – 34 | 78 – 97  3 – 22 |
| 70 | 38 - 62 | 48 – 72  28 – 52 | 58 – 80  20 – 42 | 69 – 89  21 – 31 | 80 – 96  4 – 20 |
| 100 | 40 - 60 | 50 – 70  30 – 50 | 60 – 79  21 – 40 | 71 – 87  13 – 29 | 82 – 95  5 – 18 |
| 200 | 43 - 57 | 53 – 67  33 – 47 | 63 – 76  24 – 37 | 74 – 85  15 – 26 | 85 – 94  6 – 15 |

**Further information on the questionnaire**

The survey included dichotomous responses as well as categorical variables with more than two response options which allowed the participants to select multiple choices. Questions related to symptoms and diagnoses were provided in a free-text format, allowing respondents to list up to five complaints and four diagnoses, respectively. Patient satisfaction with the different treatment modalities was assessed using a five-point Likert scale (from 1 = not very helpful to 5 = very helpful). (Pharmaco)therapy was reported in a free-text format and categorized according to the substance groups (analgesics (non-opioid and opioid) and the type of administration (“daily” vs “if required”).
