# Supplementary material for: Health care supply in patients with Ehlers-Danlos syndromes and generalized hypermobility spectrum disorder: a German perspective
Source: Orphanet J Rare Dis. 2025 Aug 16;20:436. doi: 10.1186/s13023-025-03937-4 (PMC12358066; doi:10.1186/s13023-025-03937-4)
Supplement: Supplementary file 2 — Supplementary Material 2 [file 13023_2025_3937_MOESM2_ESM.docx]

## **Additional file 2.** Complaints reported as “severely affecting daily activities” in our cohort.

|  | **Total** |  | **Monogenetic** | **hEDS/ HSD** |
| --- | --- | --- | --- | --- |
|  | **n (%)** | **95% CI** | **n (%)** | **n (%)** |
|  | 98 (100) |  | 18 (100) | 80 (100) |
| Musculoskeletal |  |  |  |  |
| Pain (musculoskeletal) | 59 (60.2) | [50.3, 69.5] | 13 (72.2) | 46 (57.5) |
| Joint instability | 38 (38.8) | [29.6, 48.6] | 7 (38.9) | 31 (38.8) |
| Other Orthopedic Disorders | 36 (36.7) | [27.7, 46.6] | 7 (38.9) | 29 (36.3) |
| Gastrointestinal | 35 (35.7) | [26.8, 45.5] | 4 (22.2) | 31 (38.8) |
| Fatigue | 32 (32.7) | [24.0, 42.3] | 5 (27.8) | 27 (33.8) |
| Autonomic nervous system | 28 (28.6) | [20.3, 38.0] | 3 (16.7) | 25 (31.3) |
| Pain (general) | 24 (24.5) | [16.8, 33.7] | 3 (16.7) | 21 (26.3) |
| Cutaneous and tissue disorders | 14 (14.3) | [8.4, 22.2] | 4 (22.2) | 10 (12.5) |
| Headaches or migraines | 11 (11.2) | [6.1, 18.6] | 2 (11.1) | 9 (11.3) |

Note. multiple references may occur; totals do not add up to 100%.; hEDS, hypermobile Ehlers-Danlos syndrome; HSD, hypermobility spectrum disorder; CI, confidence interval, calculated using the Clopper-Pearson method.

Autonomic nervous system included postural orthostatic tachycardia syndrome; Joint instability, included dislocation,

subluxation, temporomandibular joint; Other Orthopedic Disorders, included spine disorders, scoliosis, hip dysplasia,

foot anomalies.
