# Supplementary material for: Health care supply in patients with Ehlers-Danlos syndromes and generalized hypermobility spectrum disorder: a German perspective
Source: Orphanet J Rare Dis. 2025 Aug 16;20:436. doi: 10.1186/s13023-025-03937-4 (PMC12358066; doi:10.1186/s13023-025-03937-4)
Supplement: Supplementary file 3 — Supplementary Material 3 [file 13023_2025_3937_MOESM3_ESM.docx]

## **Additional file 3.** Comorbidities self-reported in our cohort

|  | **Total** |  | **Monogenetic** | **hEDS/ HSD** |
| --- | --- | --- | --- | --- |
|  | **n (%)** | **95% CI** | **n (%)** | **n (%)** |
|  | 98 (100) |  | 19 (100) | 79 (100) |
| Patients with comorbidities ^*^ | 77 (78.6) | [69.1, 86.2] | 9 (47.4) | 68 (86.1) |
| 1-2 comorbidities | 18 (18.4) | [11.3, 27.5] | 4 (21.1) | 14 (17.7) |
| 3 comorbidities | 15 (15.3) | [8.8, 24.0] | 1 (5.3) | 14 (17.7) |
| At least 4 comorbidities ^*^ | 44 (44.9) | [34.8, 55.3] | 4 (21.1) | 40 (50.6) |
| Most common comorbidities |  |  |  |  |
| Asthma | 21 (21.4) | [13.8, 30.9] | 2 (10.5) | 19 (24.1) |
| Depression | 21 (21.4) | [13.8, 30.9] | 2 (10.5) | 19 (24.1) |
| Irritable bowel syndrome | 14 (14.3) | [8.0, 22.8] | - | 14 (17.7) |
| POTS | 13 (13.3) | [7.3, 21.6] | 1 (5.3) | 12 (15.2) |
| Small Fiber Neuropathy | 10 (10.2) | [5.0, 18.0] | - | 10 (12.7) |
| MCAS | 10 (10.2) | [5.0, 18.0] | - | 10 (12.7) |
| Migraine | 9 (9.2) | [4.3, 16.7] | 1 (5.3) | 8 (10.1) |
| Arthritis | 8 (8.2) | [3.6, 15.5] | 1 (5.3) | 7 (8.9) |
| Chronic pain | 8 (8.2) | [3.6, 15.5] | 2 (10.5) | 6 (7.6) |
| PTSD | 8 (8.2) | [3.6, 15.5] | - | 8 (10.1) |

Note. hEDS, hypermobile Ehlers-Danlos syndrome; HSD, hypermobility spectrum disorder; CI, confidence interval, calculated using the Clopper-Pearson method; POTS, postural orthostatic tachycardia syndrome; MCAS, mast cell activation syndrome; PTSD, post-traumatic stress disorder.

Number of patients with self-reported diagnoses; multiple references possible as free text, which was coded; codes were not mutually exclusive. Numbers for most common comorbidities do not sum to the total because of missing data.

*p-values by two-sided Fisher’s exact test: patients with comorbidities (p **< .001**), ≥4 comorbidities (p = **.023**).
